# Up or down? Reading direction influences vertical counting direction in the horizontal plane – a cross-cultural comparison

**DOI:** 10.3389/fpsyg.2015.00228

**Published:** 2015-03-10

**Authors:** Silke M. Göbel

**Affiliations:** Department of Psychology, University of YorkYork, UK

**Keywords:** mental number line, grounded cognition, SNARC, spatial–numerical association, children, physical world account

## Abstract

Most adults and children in cultures where reading text progresses from left to right also count objects from the left to the right side of space. The reverse is found in cultures with a right-to-left reading direction. The current set of experiments investigated whether vertical counting in the horizontal plane is also influenced by reading direction. Participants were either from a left-to-right reading culture (UK) or from a mixed (left-to-right and top-to-bottom) reading culture (Hong Kong). In Experiment 1, native English-speaking children and adults and native Cantonese-speaking children and adults performed three object counting tasks. Objects were presented flat on a table in a horizontal, vertical, and square display. Independent of culture, the horizontal array was mostly counted from left to right. While the majority of English-speaking children counted the vertical display from bottom to top, the majority of the Cantonese-speaking children as well as both Cantonese- and English-speaking adults counted the vertical display from top to bottom. This pattern was replicated in the counting pattern for squares: all groups except the English-speaking children started counting with the top left coin. In Experiment 2, Cantonese-speaking adults counted a square array of objects after they read a text presented to them either in left-to-right or in top-to-bottom reading direction. Most Cantonese-speaking adults started counting the array by moving horizontally from left to right. However, significantly more Cantonese-speaking adults started counting with a top-to-bottom movement after reading the text presented in a top-to-bottom reading direction than in a left-to-right reading direction. Our results show clearly that vertical counting in the horizontal plane is influenced by longstanding as well as more recent experience of reading direction.

## INTRODUCTION

Spoken language affects various aspects of number processing and arithmetic. For example, the way number words are constructed differs between languages. The complexity of number word construction influences early counting, arithmetic and place-value understanding (Dowker et al., 2008; Siegler and Mu, 2008; Zuber et al., 2009) and inconsistencies between the Arabic notation and number word construction (e.g., number word inversion) lead to disadvantages in symbolic number processing (Pixner et al., 2011) and affects symbolic arithmetic (Göbel et al., 2014b). Written language practices also affect numerical cognition. For example, the direction of reading and writing within a culture can influence number processing (Göbel et al., 2011). The current paper focuses on the influence of reading direction on the direction of counting by comparing the counting of children and adults in the United Kingdom (UK) to children and adults from Hong Kong (HK).

Most Western adults and children count objects horizontally from left to right (Opfer and Thompson, 2006; Opfer et al., 2010; Shaki et al., 2012). This counting bias might be yet another instantiation of the mental number line, a common spatial–numerical association (SNA) of small numbers with left and larger numbers with right space (Fischer and Brugger, 2011). Evidence for a mental number line with a left-to-right direction comes from a large body of research investigating the spatial–numerical association of response codes (SNARC) effect: in parity judgment participants are consistently faster to respond with left responses to smaller and right responses to larger numbers (Dehaene et al., 1993; for a review see Wood et al., 2008).

Interestingly, several recent studies have reported the existence of horizontal SNAs already in young infants and animals: newly hatched and 3-day-old chicks have a tendency to associate large numbers with the right side of space (Rugani et al., 2014, 2015), chimpanzees and rhesus monkeys associate smaller numbers with starting on the left side of space (Adachi, 2014; Drucker and Brannon, 2014) and 7-month-old infants prefer displays that increase in magnitude to be shown from left to right (de Hevia et al., 2014). These findings point toward a biological predisposition for early horizontal SNAs (Rugani et al., 2010, 2011). Hemispheric lateralization could account for an advantage in processing the left hemispace: an early right hemispheric dominance in visuo-spatial tasks might lead to a stronger allocation of attention to the left hemifield (Mesulam, 1990). Combined with a preference for increasing sequences (Macchi Cassia et al., 2012) this hemispheric asymmetry could provide the early building blocks of a left-to-right SNA. Some evidence for this *hemispheric asymmetry account* comes from adult neglect patients (Heilman and Van Den Abell, 1980): after damage to their right parietal lobe they typically show a rightward shift in line and number bisection (Umiltà et al., 2009), pointing toward a role of the right parietal lobe in attending toward left space (Göbel et al., 2006). However, the hemispheric asymmetry account cannot explain why illiterate adults showed no significant SNARC effect (Zebian, 2005) and no preference for a particular horizontal counting direction (Shaki et al., 2012). Studies on illiterate adults provide strong support for an alternative account for horizontal SNAs: *the reading direction account.*

This account suggests that the direction of the mental number line is shaped by the culturally dominant reading direction. Already in the first paper on the SNARC effect Dehaene et al. (1993) provided evidence that the size and possibly the direction of this effect might be related to reading direction. They investigated the SNARC effect in a group of participants who originated from a right-to-left reading culture (Iran) but were living in a left-to-right reading culture (France) at the time of testing. The strength of their SNARC effect was correlated with the length of time spent in the left-to-right reading culture. In addition, there is evidence for a reversal in the direction of the SNARC effect and the dominant counting direction in cultures with a right-to-left reading direction. Arab participants who read from right to left show a reversed SNARC effect: they are faster to respond to small numbers with a right and to larger numbers with a left response (Zebian, 2005; Shaki et al., 2009). Similarly, the majority of Arab adults and children count from right to left (Shaki et al., 2012). In summary, those findings are most convincingly explained by the reading direction account.

Taking this account a step further, the reading direction account predicts a vertical mental number line in cultures reading from top to bottom. At this point it is important to clarify that the term vertical is used in two ways: in a two-dimensional context, for example when reading a page of a book, the vertical axis refers to the axis perpendicular to the horizontal axis. However, in 3D the true vertical axis is perpendicular to the horizontal plane. Surprisingly little research has investigated SNAs in the vertical dimension and most research on vertical SNAs so far has focused on the vertical axis in the horizontal plane (see Hartmann et al., 2014).

During number processing some people automatically activate visuo-spatial images of number lines (so called number forms) that are stable over time and highly individual. Already in an early description, many of these forms (Galton, 1880, Figures 2, 4, 6 and 8) progress not only from left to right but also from bottom to top. In a study of 15 Belgium university students with number forms, nine number forms progressed from the bottom up and only one from top to bottom (Seron et al., 1992). Sagiv et al. (2006) classified the direction of number forms of 114 Scottish synaesthetes and 311 controls without synaesthesia as either left-to-right, right-to-left, bottom-to-top, or top-to bottom exclusively. The majority was classified as left-to-right, but 11% of the synaesthetes’ number forms and 23% of the controls’ number forms progressed bottom-to-top and none showed a top-to-bottom direction. This suggests, at least in individuals with number forms, a predominant vertical association of small numbers with bottom and larger numbers with top space.

Research suggests that this vertical association is not specific to just people with explicit number forms. Schwarz and Keus (2004) found a truly vertical SNARC effect in Dutch participants: eye movements to a bottom response location started earlier for smaller than larger numbers while eye movements to a top response location begun earlier for larger than smaller numbers. Further, a vertical SNARC effect has been found in Belgium, American, German, and Israeli participants (Gevers et al., 2006; Müller and Schwarz, 2007; Holmes and Lourenco, 2012; Shaki and Fischer, 2012; Hartmann et al., 2014). The majority of these studies (Gevers et al., 2006; Müller and Schwarz, 2007; Shaki and Fischer, 2012) used vertical responses in the horizontal plane, i.e., close and far response buttons. However, two of these studies (Holmes and Lourenco, 2012; Hartmann et al., 2014) used a truly vertical response button arrangement and found that participants were faster to respond to small numbers with bottom hand responses and large numbers with top hand responses.

Those findings support the idea of a vertical dimension of number magnitude with increasing magnitude from bottom to top. This direction of the vertical SNA is opposite to predictions from the reading direction account. At first, one might think that in Western participants the dominant reading direction is from left to right and thus neutral with respect to the vertical dimension. However, given that most reading and writing in adults involves more than one line of text, reading and writing have a secondary direction: line by line, from the top to the bottom of a page. A strong version of the reading direction account thus proposes that the secondary reading direction (top-to-bottom) should also influence the direction of the SNA and lead to an association of small numbers with top and larger numbers with bottom space. However, I suggest an alternative: a weaker version of the reading direction account proposes that only the dominant reading direction is affecting SNAs and not the secondary reading direction. This weaker version can account for the horizontal SNA, but is silent with respect to the vertical SNA found in left-to-right and right-to-left reading cultures. Interestingly, this hints at possibly different mechanisms underlying horizontal and vertical SNAs.

Vertical associations might reflect experience with the physical world (Lakoff and Núñez, 2000; Gevers et al., 2006). In the physical world magnitude is often associated with higher up in the vertical dimension: more water in a glass is indicated by a higher level, higher buildings and trees and taller people extend more upward than smaller ones. If the association between number magnitude and vertical space is mainly driven by experiences in the physical world (*the physical world account*) then the association of small numbers with the bottom and larger numbers with the top space should be found independent of cultural context. In Fischer’s (2012) terminology, this physical world account is a grounded theory (Barsalou, 2008, p. 162) based on “invariants in the physical world”. Support for this account comes for example from a study by Lachmair et al. (2014). In a lexical decision task, after being primed with small numbers, participants were significantly faster to respond to words that are normally associated with lower vertical space (e.g., submarine). In contrast, words associated with upper vertical space (e.g., eagle) were significantly faster responded to when the prime was a large number.

Research on vertical SNAs in Japan, a culture with a dominant reading direction from top to bottom, strongly supports the physical world account. Ito and Hatta (2004) asked 50 Japanese undergraduate students to place 0–9 on a vertical line. The majority (76%) placed ascending numbers from bottom to top and only 18% used a top-to-bottom arrangement, arguing against a dominant influence of vertical reading direction. When Japanese participants performed a vertical SNARC task with response buttons in the horizontal plane they also showed the same association as Western participants: Japanese participants were faster to respond to smaller numbers with bottom than top responses and to larger numbers with top than bottom responses. The direction of their vertical SNAs was opposite to their reading direction and in line with the physical world account.

A study with Taiwanese participants, however, showed that whether reading direction influences the SNARC effect in the horizontal and vertical dimension might depend on the number format used in the task. There are three numerical notations in Taiwan: (1) Arabic digits (e.g., 1), (2) Chinese number words in the simple form (e.g.,), (3) Chinese number words in the complex form (e.g.,). Hung et al. (2008) tested the horizontal and vertical SNAs of these three notations in Taiwanese participants. Arabic digits are typically printed horizontally in text in Taiwan, while Chinese number words appear more often in vertical text with a top-to-bottom directionality. For Arabic digits they found a significant horizontal SNARC effect with faster left than right responses for smaller digits and faster right than left responses for larger digits, but there was no significant horizontal SNARC effect for Chinese number words. In contrast, the vertical association between numbers and space was only significant for the Chinese number words in the simple form, but not for Arabic digits or Chinese number words in the complex form. Chinese number words in the simple form were responded to faster with top than bottom responses for small numbers and faster with bottom than top responses for large numbers. This suggests that the association between number and space is not hardwired, but flexible (Bächtold et al., 1998; Ristic et al., 2006; Fischer et al., 2009, 2010) and can adapt rapidly to a different context. In Fischer’s (2012) terminology, this speaks for the situatedness of SNAs. Furthermore, it was the dominant reading direction associated with the specific number notation used in the task that predicted the specific direction of the SNA. The different results found for Chinese number words in the simple and in the complex form suggest that in order to influence SNAs the association between notation and reading direction needs to be strong and firmly established. Chinese number words in the complex form probably did not influence the direction of SNA, because they are less frequent than Arabic digits and Chinese number words in the simple form and do not strongly evoke a reading context.

In summary, SNAs also exist in the vertical dimension. The most common association seems to be along a mental number line with numbers with increasing magnitude going from bottom to top space. Reading direction possibly can influence this association under certain conditions.

The first aim of the current study was to investigate whether reading direction influences the direction of vertical counting in the horizontal plane. To the best of my knowledge, this has not yet been investigated. An explicitly spatial-numerical task (object counting) was chosen rather than the implicit, more commonly used SNA task of number judgment because we have shown that reading direction influences the horizontal counting direction (Shaki et al., 2012). Furthermore, so far no study has directly investigated the effect of reading direction on implicit SNA tasks in young children while there is evidence from our own work (Göbel et al., 2014a) that recent reading observation, even in preliterate children, can change their horizontal counting direction. Investigating vertical counting was logically the next step. I chose two groups of participants with different reading experiences: (1) participants with a dominant reading direction from left to right and a secondary reading direction from top to bottom (UK), (2) participants with mixed dominant and secondary reading direction (Hong Kong [HK]). The majority of text in books and newspapers in HK is printed from left to right with a secondary reading direction from top to bottom. A visible minority of text, however, is presented in top-to-bottom direction with the secondary reading direction going from right to left ^1^. I was interested in the effect of both dominant and secondary reading direction on the direction of counting. The second aim was to investigate whether the amount of reading (and writing) experience influences the strength of the association. We therefore tested both children and adults. Children were beginning readers and had thus much smaller experience with the cultural direction of reading and writing than adults. Third, given that there might be different mechanisms underlying horizontal and vertical SNAs I was interested in whether there is a hierarchy in the association of number and space. For example, are horizontal SNAs more dominant than vertical SNAs? We tested this by asking participants to count objects in a display with balanced vertical and horizontal dimensions (a square of objects). Lastly, we were interested in how flexible those spatial biases are. Thus, in Experiment 2 we manipulated the most recent reading experience direction (left-to-right or top-to bottom) and investigated whether the most recent reading experience shows an immediate effect on counting direction.

## EXPERIMENT 1

**FIGURE 1:**
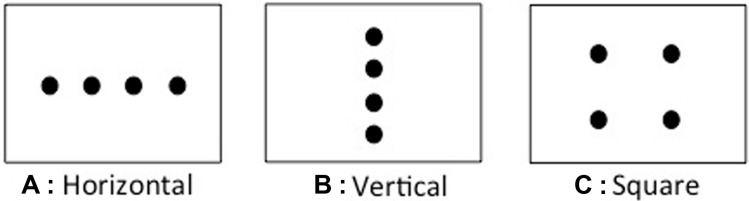
**Schematic of the counting displays used in Experiment 1 (not drawn to scale).** **(A)** Horizontal array, **(B)** vertical array, **(C)** square array.

Adults and children in the UK and in HK were asked to count objects in three differently arranged displays: a horizontal, a vertical, and a square display (**Figure 1**). In line with their dominant reading direction, we expected the majority of all participants to count the horizontal array from left to right. With respect to counting the vertical array, the strong reading direction account predicts that all participants will count from top to bottom, while the weak reading account predicts no preference for a specific vertical counting direction in UK participants, but a top-to-bottom preference for HK participants. We expected the children to show this pattern less strongly than the adults due to their limited experience with reading and writing. The physical world account, in contrast, predicts that most participants will count the vertical array from bottom to top. For counting objects in the square arrangement there are two factors of interest: first, the starting position and second, the direction of the first movement. The reading account predicts a top left starting position and a first movement from left to right for all participants. The physical world account predicts a bottom starting position and a first movement from bottom to top, but is neutral with respect to left or right side.

### MATERIALS AND METHODS

#### Participants

All British participants (80 children, 100 adults) were native English speakers brought up in the UK. All HK-Chinese participants (94 children, 99 adults) were native Cantonese speakers brought up in HK. British 4-and 5-year-old children were tested with parental consent in nurseries and primary schools in North Yorkshire, Greater Manchester, and Shrewsbury. HK-Chinese 4-and 5-year-old children were tested with parental consent in kindergartens in HK. All adult participants gave written consent. British adults were tested in the UK, HK-Chinese adults in HK. Data for left-handed children and adults were excluded. I am reporting data for the remaining 71 British children (mean age = 4.44 years, SD = 0.50, 33 female, 38 male), 85 HK-Chinese children (mean age = 4.82, SD = 0.38, 51 female, 34 male), 90 British adults (18–94 years, mean age = 48.07 years, SD = 21.64, 58 female, 32 male) and 99 HK-Chinese adults (18-83 years, mean age = 32.98, SD = 14.80, 59 female, 40 male). The study was approved by the Ethics Committee, Department of Psychology, University of York.

#### Materials

Twelve golden plastic coins (diameter = 3.5 cm) and three rectangular mats (40 cm × 30 cm, landscape) were used to create three counting displays (**Figure 1**). For the horizontal display four coins were placed horizontally in a linear array onto the mat, equidistant (4.0 cm) from each other with the two outer coins placed at about 6.3 cm from the side edges of the mat and all coins at about 13.3 cm from the top and bottom edges of the mat. In the vertical display four coins were placed flat on the mat, vertically in a linear array equidistant (3.0 cm) from each other with the coins placed at about 18.3 cm from the side edges and at about 3.5 cm from the top and bottom edges. In the square display four coins were placed into a 2 by 2 square, with about 8.0 cm between each coin, with the outer edges of the square arrangement at about 12.5 cm from the left and right edges of the mat and at about 7.5 cm from the top and bottom edges.

#### Procedure

All three stimuli sets (horizontal, vertical, and square display) were prepared before testing and covered with DIN A3 sheets of paper. Participants were tested individually in a quiet room. HK-Chinese participants were tested in Cantonese, by a native Cantonese speaker. British participants were tested in English, by a native English speaker. Stimuli were present lying flat on the table at which the participant was seated, centrally in front of the participant, and covered. The first stimulus set was then presented by lifting off the cover. Participants were asked, “Can you please point to each of the coins for me and count aloud how many there are?” No demonstration was given, and participants’ order of counting was recorded by the experimenter. The instruction was repeated twice again with the next two stimulus sets. Next, handedness was tested. Children were asked to draw a picture of a sun. Adults filled out the Edinburgh Handedness Questionnaire (Oldfield, 1971). In addition, children in HK were asked to write three age-appropriate characters (big, small, mother). At the end participants were thanked, children were praised, and received a sticker. All participants counted the horizontal, vertical, and square displays. The order of the presentation of the three displays and the seating position of the experimenter (to the left or right of the participant) was counterbalanced between participants.

### RESULTS

#### Horizontal array

**FIGURE 2:**
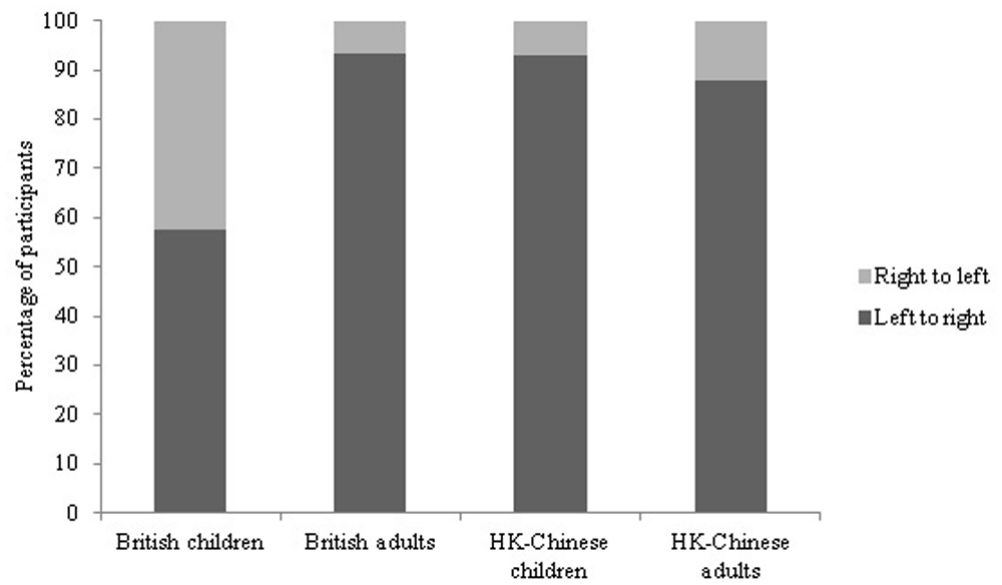
**Percentage of participants counting the horizontal display left to right vs. right to left**.

As can been seen in **Figure 2**, the majority of all participants counted the horizontal display from left to right (57.7% of the British children, 93.3% of the British adults, 92.9% of the HK-Chinese children, and 87.9% of the Chinese adults, Supplementary Table S1). The difference between the number of participants counting left to right and right to left was significant for British adults (χ^2^ = 67.6, df = 1, *p* < 0.01), HK-Chinese children (χ^2^ = 62.69, df = 1, *p* < 0.01) and HK-Chinese adults (χ^2^ = 56.82, df = 1, *p* < 0.01). For the British children, there was no significant preference in counting direction (χ^2^ = 1.70, df = 1, *p* = 0.19). Significantly more British adults counted from left to right than British children (χ^2^ = 29.0, df = 1, *p* < 0.001). There was no significant difference in counting frequency between the HK-Chinese children and adults (χ^2^ = 1.33, df = 1, *p* = 0.25) or between the British adults and HK-Chinese adults (χ^2^ = 1.63, df = 1, *p* = 0.20). Although more British 5-year-olds (67.7%) than 4-year-olds (50.0%) counted left to right this difference did not reach significance (χ^2^ = 2.25, df = 1, *p* = 0.13) and there was no effect of age on the horizontal counting direction for HK-Chinese children either (χ^2^ = 1.38, df = 1, *p* = 0.24). There was no effect of order, experimenter location or gender on the frequency of horizontal counting direction in any group (all *p*s > 0.05).

#### Vertical array

**FIGURE 3:**
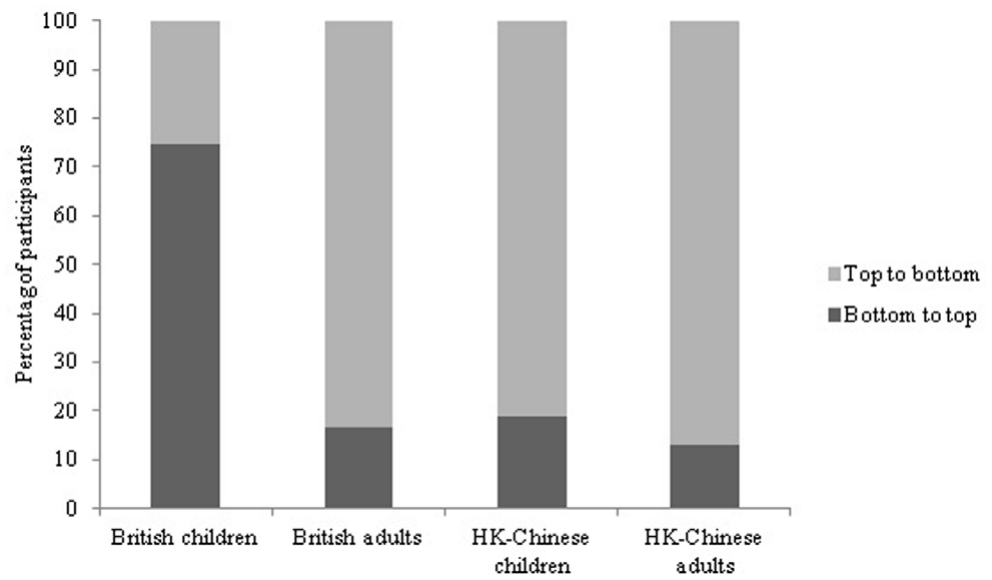
**Percentage of participants counting the vertical display top to bottom vs. bottom to top**.

Significantly more British children (74.6%) counted from bottom to top than from top to bottom (25.4%; χ^2^ = 17.25, df = 1, *p* < 0.01). In contrast, the majority of British adults (83.3%, χ^2^ = 40.00, df = 1, *p* < 0.01), HK-Chinese children (81.2%, χ^2^ = 33.05, df = 1, *p* < 0.01) and HK-Chinese adults (86.9%, χ^2^ = 53.83, df = 1, *p* < 0.01) counted from top to bottom (see **Figure 3**, Supplementary Table S1). The counting patterns between British children and adults were significantly different (χ^2^ = 54.7, df = 1, *p* < 0.01). There was no significant difference in counting frequency neither between the HK-Chinese children and adults (χ^2^ = 1.12, df = 1, *p* = 0.29) nor between the British adults and the HK-Chinese adults (χ^2^ = 0.47, df = 1, *p* = 0.49), but there was a significant difference between HK-Chinese children and British children (χ^2^ = 48.90, df = 1, *p* < 0.001). There was no significant difference in counting preference between the 4-and 5-year-old children neither for the British children nor for the HK-Chinese children (all *p*s > 0.05). Gender did not affect the counting direction (all *p*s > 0.05). While there was no effect of order or experimenter location for British or Chinese adults (all *p*s > 0.05), order had a significant effect for both British, and HK-Chinese children. For British children significantly more children counted top to bottom when the vertical array came after the square (50.0%) than when it came before the square array (15.75%; χ^2^ = 8.94, df = 1, *p* < 0.001). The same pattern was observed for the HK-Chinese children: significantly more Chinese children counted top to bottom when the vertical array came after the square array (90.24%) than when it came before the square array (72.72%; χ^2^ = 4.26, df = 1, *p* = 0.04). Experimenter location did not affect counting direction for the HK-Chinese children (χ^2^ = 0.253, df = 1, *p* = 0.62). However, significant more British children with the experimenter sitting on their right side (36.3%) counted top to bottom than British children with the experimenter sitting on their left side (15.8%, χ^2^ = 3.95, df = 1, *p* = 0.047).

#### Square array

The data for one 5-year-old British child were excluded from the data analysis because he moved diagonally when counting the coins in the square. The majority of the British children started to count either on the bottom left (31.4%) or right (41.4%) coin. All other groups showed a clear preference to start counting with the top left coin (British adults: 88.9%, HK-Chinese children: 71.8%; HK-Chinese adults: 81.8%; see **Table 1**).

**Table 1:** Number of participants by starting position and direction of first movement for counting the square display for Experiment 1.

	Starting position				First movement			
	Left		Right		Horizontal		Vertical	
Group	Top	Bottom	Top	Bottom	Left–right	Right–left	Bottom–top	Top–bottom
**British**
Children	13	22	6	29	24	15	26	5
Adults	80	2	3	5	77	3	4	6
**HK-Chinese**
Children	61	7	7	10	54	5	11	15
Adults	81	3	12	3	76	4	2	17

***Vertical starting position.*** Significantly more British children (72.9%) started counting the square on one of the two bottom coins than on one of the two top coins (27.1%; χ^2^ = 14.63, df = 1, *p* < 0.01). In contrast, the majority of British adults (92.2%, χ^2^ = 64.18, df = 1, *p* < 0.01), HK-Chinese children (80.0%, χ^2^ = 30.60, df = 1, *p* < 0.01) and HK-Chinese adults (93.9%, χ^2^ = 76.46, df = 1, *p* < 0.01) started at a top coin. The counting patterns between British children and British adults (χ^2^ = 72.2, df = 1, *p* < 0.01) as well as between the British children and the HK-Chinese children (χ^2^ = 43.6, df = 1, *p* < 0.001) were significantly different. Although overall most HK-Chinese children and adults counted the square starting from a top coin, there were significantly more HK-Chinese children (20.0%) who started counting the coins in the square from a bottom coin than HK-Chinese adults (6.1%, χ^2^ = 8.12, df = 1, *p* < 0.01). There was no significant difference in counting preference between the British adults and the HK-Chinese adults (χ^2^ = 0.22, df = 1, *p* = 0.64).

***Horizontal starting position.***All groups except the British children showed a clear preference to start counting the coins in the square on the left side (British adults: 91.1%, χ^2^ = 60.84, df = 1, *p* < 0.01; HK-Chinese children: 80.0%, χ^2^ = 30.60, df = 1, *p* < 0.01, HK-Chinese adults: 84.8%, χ^2^ = 49.5, df = 1, *p* < 0.01). For British children there was no significant difference between the number of children starting counting the coins in the square on the left (50.0%) versus on the right side (50.0%, χ^2^ = 0, df = 1, *p* = 1.00). The counting patterns between British children and British adults (χ^2^ = 33.9, df = 1, *p* < 0.001) as well as between the British children and the HK-Chinese children (χ^2^ = 15.5, df = 1, *p* < 0.001) were significantly different. There was no significant difference in counting preference neither between the HK-Chinese children and HK-Chinese adults (χ^2^ = 0.748, df = 1, *p* = 0.39) nor between British adults and the HK-Chinese adults (χ^2^ = 1.73, df = 1, *p* = 0.19).

***First movement*.** As expected, the first movement when counting the coins in the square was horizontal for most British adults (88.9%, χ^2^ = 54.4, df = 1, *p* < 0.01), HK-Chinese children (69.4%, χ^2^ = 12.81, df = 1, *p* < 0.01) and HK-Chinese adults (80.8%, χ^2^ = 37.59, df = 1, *p* < 0.01) and the majority moved from left to right (British adults: 85.6%, HK-Chinese children: 63.5%, HK-Chinese adults: 76.8%). In contrast, for British children there was no significant preference for moving horizontally (55.7%) or vertically (44.2%; χ^2^ = 0.914, df = 1, *p* = 0.34) first. This pattern was significantly different from the counting pattern for British adults (χ^2^ = 22.7, df = 1, *p* < 0.001) and for HK-Chinese adults (χ^2^ = 12.4, df = 1, *p* < 0.001) and approaching a significant difference to the counting pattern for HK-Chinese children (χ^2^ = 3.10, df = 1, *p* = 0.08). 34.3% of British childrens’ first movement was from left to right, 21.4% from right to left, 37.2% from bottom to top and 7.1% from top to bottom. For more details, please see **Table 1**.

***Experimenter seating position, order, gender, and children’s age.*** There were no significant differences between the square counting patterns of 4 and 5 year olds for the British or the HK-Chinese children and no effect of gender (all *p*s > 0.05). For British children and adults as well as for HK-Chinese adults experimenter seating position and order of the square array did not significantly affect their counting behavior (all *p*s > 0.05). However, for the HK-Chinese children significantly more children (29.5%) started counting at the bottom than the top when the square was presented after the vertical display than when it was presented before (9.8%, χ^2^ = 5.19, df = 1, *p* = 0.02). Equally, their first movement was significantly more likely to be vertical when the square display was presented after the vertical array (40.9%) than when it was presented before the vertical array (19.5%, χ^2^ = 4.58, df = 1, *p* = 0.03). In addition, significantly more HK-Chinese children started counting the square on the right side when the experimenter was sitting on their left (32.6%) than when she was sitting on their right side (7.7%, χ^2^ = 8.58, df = 1, *p* < 0.01). All other effects of order and experimenter location were non-significant.

### DISCUSSION

Overall, results from Experiment 1 broadly support the reading direction account. As predicted, there was a preference to count the horizontal array from left to right. For British children this preference was present, but not statistically significant. For all other groups the left-to-right preference was statistically significant supporting the reading direction account of horizontal SNAs. At first, the finding that British children did not show a significant preference of horizontal counting direction seems to be at odds with previous findings of a left-to-right SNA in 3-6-year-old Western children (Patro and Haman, 2012; Shaki et al., 2012; Knudsen et al., in press). However, previous studies have shown that horizontal SNAs can only be elicited in young children under certain conditions (Hoffmann et al., 2013) and that they are less pronounced than in older children or even absent (Berch et al., 1999; Van Galen and Reitsma, 2008). In addition, the percentage of children counting left to right found in our study (57.7%) is comparable to a previous study in which 60.7% of UK pre-school children showed a preference for counting from left to right (Shaki et al., 2012). In this study the preference for counting from left to right in UK children increased significantly from preschool into school age lending further support to the reading direction account.

For the vertical array, in line with the strong reading direction account, the majority of adults and HK-Chinese children in our study counted the coins from the top to the bottom. British children, however, showed a significant preference to count from bottom to top. A similar pattern was observed for counting the square array: while most British adults, HK-Chinese adults and HK-Chinese children started counting with the top left coin, British children preferred to start counting with a bottom coin with no preference for either the left or right bottom coin. In summary, the reading direction account explains the findings from British adults, HK-Chinese adults, and HK-Chinese children.

In contrast, the counting patterns of British children are in line with the physical world account. Although we chose the same age groups for British and HK-Chinese children, HK-Chinese children start being taught to write (and read) in Chinese from around age 3 (Curriculum Development Council, 2006). This is much earlier than for British children. I suggest that the differences in counting patterns between the British and the HK-Chinese children are explained by the fact that the two groups of children were not matched on reading (and writing) experience. For young children with little reading skill the experience of magnitude in the physical world might dominate their vertical SNAs and the culturally dominant reading direction only begins to shape their SNAs with increasing exposure to and experience of reading and writing. There are two aspects of our data that support this conclusion: first, although HK-Chinese children showed a clear preference to count the square starting at the top left coin, significant more HK-Chinese children than HK-Chinese adults started counting from a bottom coin, showing some residual pattern in line with the physical world account. Second, although British children did not show a statistically significant preference for a particular reading direction in the horizontal direction as predicted by the physical world account, descriptively more British children (57.7%) counted from left to right than right to left. I argue that this might be a hint of the emergence of the effect of reading direction on horizontal counting direction in British children.

## EXPERIMENT 2

Experiment 2 tested the flexibility of the counting pattern. Previous research (Bächtold et al., 1998; Ristic et al., 2006; Fischer et al., 2009, 2010) has shown that the SNARC effect is flexible and can be altered easily by short spatial experiences. For example, in a study by Shaki and Fischer (2008) bilingual Russian-Hebrew speakers showed a significant horizontal SNARC effect after reading a Russian text for 10 min (written in Cyrillic, reading direction left-to-right), but a significantly smaller horizontal SNARC effect after reading a Hebrew text (reading direction right-to-left) for the same amount of time. Inspired by this study, we asked HK-Chinese students living in the UK to count objects arranged in a 6×6 grid after they read a horizontal or vertical text. The reading direction account predicts that overall, in line with the dominant reading direction, the majority of participants will count the objects from top left to bottom right, row by row. In addition, it is expected that more participants will count from top right to bottom left, column by column, after reading the vertical text than after reading the horizontal text. A second aim of the study was to investigate whether, similarly to Dehaene et al. (1993), the length of stay in the UK also influenced the strength of the vertical SNA.

### MATERIALS AND METHODS

#### Participants

Ninety-three right-handed native Cantonese speakers (18–25 years old, mean age = 20.63, SD = 1.27, 57 female, 36 male), brought up in HK, were tested. All had been living in the UK for less than 5 years (between 1 month and 5 years, mean years = 2.80, median = 3.00, SD = 1.43) and had given written consent. The study was approved by the Ethics Committee, Department of Psychology, University of York.

#### Materials

**FIGURE 4:**
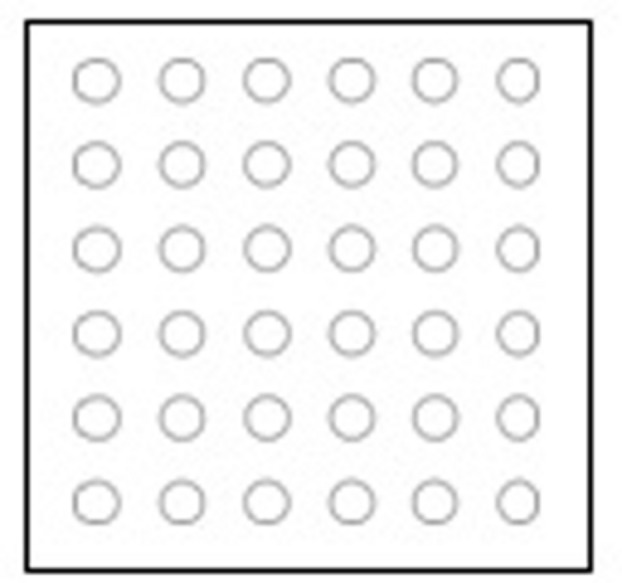
**Schematic of the 6 × 6 counting grid used in Experiment 2**.

The display consisted of 36 identical black unfilled circles (circumference = 1.6 cm) on a white piece of paper (19.2 cm by 19.2 cm). Circles were presented in a 6 by 6 grid with each circle at approximately 1.6 cm from the next circle and the outer circles at 0.4 cm from the edge (see **Figure 4**).

The reading material was a one-page text on attitudes about facing loss in Cantonese. It was taken from a website for Chinese reading comprehension (MaMa Resources, 2012; Supplementary material A, B, and C). Six comprehension questions were presented on a separate sheet of paper. There were two conditions: a vertical and a horizontal text condition. In the vertical text condition, text on all three pages (the consent form, the short article and the comprehension questions) was presented in a vertical layout. For this text presentation the reader starts at the top right corner, reading column by column top to bottom, moving from right to left for each subsequent column. In the horizontal text condition all text was presented in horizontal layout that could only be read by starting at the top left corner moving from left to right in each row, starting with the top row and reading downward row by row from the top to the bottom row. The content of the horizontally and vertically presented consent form, article, and comprehension questions was identical (see Supplementary material A and B).

#### Procedure

Participants were tested individually in the UK. Upon arrival participants were pseudorandomly allocated to either the vertical or horizontal reading condition and were tested individually in a quiet room in Cantonese by a native Cantonese speaker. Participants were asked to take a seat at the table where the stimuli had already been placed and covered before the participant arrived. Then, they were given the text to read. Subsequently they were given a sheet with comprehension questions and a pen and asked to provide the answers to the questions in writing. Participants in the vertical reading condition were given the consent form, text and comprehension questions in vertical layout, while participants in the horizontal reading condition received the consent form, text, and comprehension questions in horizontal layout. After the reading task, all participants were presented with the counting task. Participants were asked to count aloud the number of circles present on the piece of paper as quickly as possible while pointing to each circle. It was emphasized that even if it was obvious how many dots the display contained, they should still point to and count each circle. No demonstration was given, and the participants’ order of counting was recorded by the experimenter. The seating position of the experimenter (to the left or right of the participant) was counterbalanced between participants.

### RESULTS

Six participants were excluded from the data analysis because their starting position for counting was not at the top, bottom, left, or right side of the grid.

#### Starting position

All remaining participants started counting at a top position. Most participants started counting at the top left of the grid. 70 participants (80.5%) started counting on the top left and 17 participants (19.5%) started on the top right side of the grid (χ^2^ = 32.87, df = 1, *p* < 0.01). We then split participants into two groups depending on the length of stay in the UK (median split: short: less than 3 years; longer: 3 years or longer). In line with our predictions the length of time spent in the UK had a significant effect on their starting position (χ^2^ = 4.41, df = 1, *p* < 0.05, see Supplementary Table S2): although in both groups the most frequent starting position was top left, there were significantly more participants in the short stay (31.3%) than in the longer stay group (12.7%) starting counting at the top right.

**FIGURE 5:**
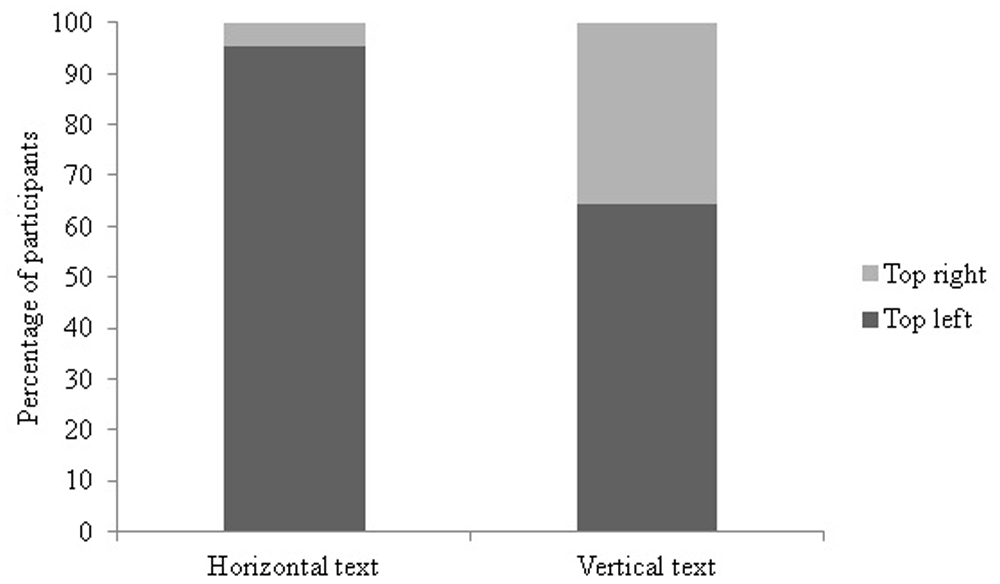
**Percentage of participants by text condition choosing top left or top right as starting position in Experiment 2**.

In line with our predictions, there was a significant effect of text direction on the starting position (χ^2^ = 13.50, df = 1, *p* < 0.01, see **Figure 5**): in the horizontal text group 95.6% of participants started counting on the top left while in the vertical text condition it was only 64.3%. For the horizontal group there was also a significant effect of length of stay in the UK on the starting position (χ^2^ = 5.76, df = 1, *p* < 0.05): none of the participants who had been in the UK three years or longer started counting on the top right, while 16.7% of the participants who arrived within the last three years did. There was no significant effect of length of stay on starting position for the vertical text group (χ^2^ = 0.31, df = 1, *p* = 0.58).

#### First movement

**FIGURE 6:**
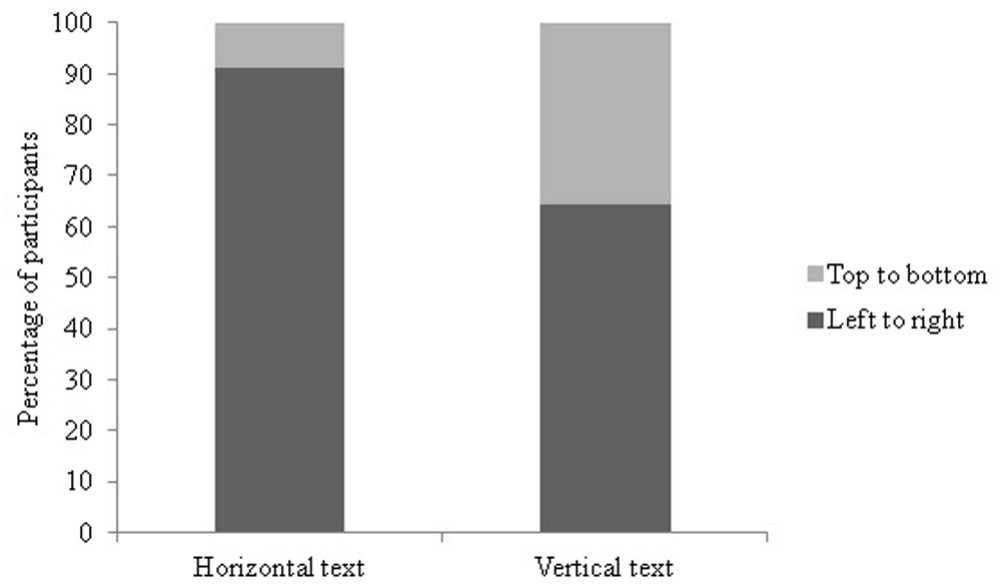
**Percentage of participants by text condition choosing left to right versus top to bottom as first movement in Experiment 2**.

As expected, the majority of participants started counting with a horizontal movement. Sixty-eight participants (78.2%) moved horizontally from their starting position, 19 (21.8%) vertically (χ^2^ = 27.60, df = 1, *p* < 0.01, see Supplementary Table S2). All of the participants moving horizontally moved from left to right, and all of the participants moving vertically moved from top to bottom. In line with our predictions there was a significant effect of text direction on the direction of the first movement (χ^2^ = 9.16, df = 1, *p* < 0.01, see **Figure 6**): in the vertical text group 35.7% of participants started counting top to bottom while in the horizontal condition it was only 8.9%. Length of stay in the UK had no significant effect on the direction of the first movement (χ^2^ = 2.63, df = 1, *p* = 0.11).

#### Gender and experimenter seating position

There were no significant effects of gender and seating position of the experimenter on starting position or first movement (all *p* > 0.09).

### DISCUSSION

Experiment 2 showed that directional reading habits dominate the counting behavior of adults. In line with their dominant reading direction, most HK-Chinese adults counted a square of circles from top left to bottom right, row by row. However, the frequency of this counting pattern was modulated by two factors: first, the most recent reading experience and second, reading experience within the last few years. Although most participants who had just read a vertical text still showed a preference for counting from top left to bottom right and row by row, significant more participants counted from top right to bottom left, column by column, after reading a vertical text than after reading the horizontal text. This is direct evidence for an influence of the most recent reading experience on the pattern of counting. Secondly, there is some evidence that the strength of this effect can be influenced by how long participants had lived in the UK: while nobody who had stayed in the UK longer than 3 years counted vertically after reading the horizontal text, two participants who had stayed in the UK less than 3 years at the time of testing did so.

## GENERAL DISCUSSION

In summary, our results strongly support the reading direction account. The majority of British and HK-Chinese participants counted the horizontal array in line with their dominant reading direction, from left to right. The vertical array in turn, they mostly counted from top to bottom, in line with their secondary reading direction, highlighting that both, horizontal and vertical, aspects of reading influence the direction of counting objects. Finally, when counting the four coins arranged in a square, the majority started on the top left coin, moved from the left to the right coin and then to the bottom left coin before ending on the bottom right coin. This pattern parallels a typically pattern of reading (Western) text on a page. Even the divergent results from the UK children fit with the reading direction account: I argue that UK children did not show a significant preference for a particular horizontal counting direction yet, because the influence of reading direction is still weak at that age due to their limited reading and writing experience. Similarly, UK children still displayed a bottom-to-top counting preference for the vertical array. I suggest that they showed this pattern because their experience with and thus the influence of the secondary reading direction is even weaker and in its absence the experience of magnitude in the physical world dominates the vertical SNA. While Experiment 1 investigated the effect of reading direction on counting by comparing groups of participant with different reading experiences, in Experiment 2 we directly manipulated the frequency of counting direction by varying the most recent reading direction. Significantly more participants counted from top right moving top to bottom, column by column, after reading a vertical text than after reading a horizontal text. These results provide direct experimental evidence of an effect of the most recent reading direction on the direction of counting. Overall, our results are best explained by the reading direction account.

However, there are alternative accounts of the origins of horizontal and vertical SNAs which will be examined in the following sections. Although horizontal SNAs seem to be weaker in younger children, several recent studies have reported the existence of horizontal SNAs in infants (de Hevia et al., 2014) and animals (Adachi, 2014; Drucker and Brannon, 2014; Rugani et al., 2014, 2015). These findings are difficult to reconcile with the reading direction account and support a biological rather than cultural account, at least for early horizontal SNAs. The hemispheric lateralization account, however, cannot explain why in our study UK children show the horizontal SNA less strongly than HK-Chinese children unless one postulates that hemispheric lateralization is stronger in HK-Chinese children than in UK children of the same age which seems unlikely. Second, the hemispheric lateralization account predicts a left-to-right counting bias in both literate and illiterate adults and does not account for the reversal in participants who read from right to left. Clearly, the hemispheric lateralization account on its own is insufficient to explain the existing data on cultural counting direction.

However, a recent study (de Hevia et al., 2014) provides a suggestion for how the hemispheric lateralization account and the reading direction account of SNAs could be reconciled (a *combination account*). They found a preference for numerical increasing sequences from left to right in 7-month-old infants. This preference was context-dependent: it was only present when infants received the increasing condition before the decreasing condition, but not when the presentation order was reversed. This suggests that there might be a biological predisposition to link numerical order to spatial directionality *and* that this early bias is easily modifiable by experiential and cultural factors such as reading direction (de Hevia et al., 2012; for an overview of other early experiential and cultural factors see Nuerk et al., 2015).

Another factor for the origin of horizontal SNAs has been suggested by Fischer and Brugger (2011): finger counting habits. Fischer (2012, p. 163) cites finger counting habits and its relationship with horizontal SNAs as an example of embodiment, “sensory and/or motor constraints of the human body,”, shaping number concepts. In an online survey of over 900 adults (Lindemann et al., 2011) the majority of Western participants reported starting counting with their left hand while the majority of Eastern participants started with their right hand. These finger counting habits are in line with the direction of their horizontal SNAs. However, this study cannot discern between two options: finger counting habits could shape the direction of horizontal SNAs or vice versa. The crucial test is whether children’s finger counting direction is predictive of their dominant object counting direction. Recent findings by (Knudsen et al., in press) suggest that the answer is likely to be ‘no’: the majority of German 6-year-old children tested started counting with fingers on their right hand, but displayed a significant preference to count objects from left to right. In addition, finger counting habits cannot explain horizontal SNAs in animals and preverbal infants.

A clear advantage of the reading direction account is that it can explain SNAs in both horizontal and vertical dimensions. Both, the finger counting habits account and the hemispheric lateralization account, cannot explain counting preferences in the vertical dimension. The preferred vertical counting direction of adults and HK-Chinese children in our study is in line with their secondary reading direction and opposite to the direction predicted by the physical world account. This is puzzling, because most studies of the vertical SNARC effect have reported a bottom-to-top orientation for increasing magnitude (Gevers et al., 2006; Müller and Schwarz, 2007; Holmes and Lourenco, 2012; Shaki and Fischer, 2012; Hartmann et al., 2014). Why did we find a clear top-to-bottom association in the vertical array for our adult participants and HK-Chinese children when most vertical SNAs have been reported to go from bottom to top? Why should a reading direction account explain vertical counting direction while the physical world account is used to explain the vertical SNARC effect? I argue that these divergent results are due to two reasons: first, in contrast to parity judgment in the SNARC experiments, counting objects is in itself a spatial and explicitly numerical activity, so with object counting we are testing explicit associations between number and space which might be different from implicit associations between number and space tested in the SNARC effect (see Nuerk et al., 2015). Second, I propose that the required spatial movement inherent in counting objects in the sagittal plane activates reading experience more strongly than choosing one of two spatial response buttons in a parity judgment task. At least in initial stages of reading, people often use their fingers to guide them when reading text on a page. Similarly, when counting objects in space, participants used their fingers to point to objects in space. I argue that object counting *per se* is a spatial activity that automatically activates magnitude and that particularly in the horizontal plane we used, at least in competent readers, this space is strongly associated with reading and writing.

In our study the group with the smallest reading and writing experience, UK children, preferred to count the vertical array from bottom to top. This association of bottom with small and top with larger magnitude can neatly be explained by the physical world account: in our daily interactions with the physical world there are many examples of experiences where ‘more is up’ (Lakoff and Núñez, 2000; Hartmann et al., 2014) with the ground level providing a natural zero (Holmes and Lourenco, 2012). Fischer (2012) argues that this is an example of grounded cognition (Barsalou, 2008). A higher mountain takes more effort and more time to climb than a smaller one. In contrast to our data, the physical world account does not predict cultural differences in vertical SNAs because the experience of the physical world is universal: the same physical principles apply independent of geographical location on our planet.

Perhaps related to experiences of magnitude in the physical world (Barsalou, 2008; Lachmair et al., 2014), we commonly encounter and use linguistic metaphors (Pecher and Boot, 2011) that associate more with higher, for example, ‘prices rise’ and ‘I’ll just turn up the volume.’ These linguistic factors have spatial consequences. After reading descriptions of magnitudes (more or less) in sentences participants were faster to respond with a top button after ‘more’ sentences and a bottom button after ‘less’ sentences (Sell and Kaschak, 2012). Even in an unrelated categorization task after judging magnitudes (few or many?) participants responded faster after a ‘many’ judgment when the item to be categorized was presented at the top of the screen than when it was presented at the bottom (Pecher and Boot, 2011). The current study does not allow us to distinguish between the physical world account and the linguistic metaphor account for vertical SNAs in inexperienced readers, because both accounts predict an association of smaller magnitude with bottom space and larger magnitude with top space. However, a study by Holmes and Lourenco (2012) indicates that the vertical direction might be less malleable by verbal (metaphorical) instruction than has been reported for the horizontal direction (e.g., Bächtold et al., 1998; Ristic et al., 2006; Fischer et al., 2010). During a parity judgment task they asked participants explicitly to think of numbers as floors of a building (bottom-to-top metaphor), as items on a shopping list (top-to-bottom metaphor) or as diving levels in a swimming pool (top-to-bottom metaphor). In all three conditions participants associated smaller numbers with bottom and larger numbers with top space. Following a physical world account for vertical SNAs, one might expect the association of small magnitude with bottom and large magnitude with top space to be strong, stable, fixed, and unaltered by instruction, because the universal physical principles on our planet (e.g., gravity) almost never change. However, our findings in Experiment 2 suggest that the vertical counting direction can be modified by recent reading direction. Also, the vertical SNARC effect can be modified by effector instruction (Müller and Schwarz, 2007) and by different number notations (Hung et al., 2008). These results speak against a fixed vertical SNA with a grounded origin and provide good evidence that vertical SNAs can also be altered by instruction and recent experiences (see Hartmann et al., 2014).

This takes us to the question of whether counting direction preferences in a truly vertical plane would be different. Most studies on vertical SNAs, including the current study, have not used a truly vertical plane but a horizontal plane with close and far locations. The horizontal plane is heavily used when reading and writing, thus favoring a situated conception. A truly vertical plane might be a better test of the physical world account for vertical associations. Two recent studies have used a truly vertical plane (Holmes and Lourenco, 2012; Hartmann et al., 2014) and reported a bottom-to-top association. To our knowledge, counting in the truly vertical direction, e.g., counting a stack of blocks has not been investigated systematically yet.

In both experiments presented here participants were asked to point to the objects and count them. On the basis of the current experiments it is not possible to exclude the possibility that pointing alone (without counting) could have resulted in spatial preferences too. Non-numerical horizontal spatial directional training can lead to changes in directional motor behavior in a visual search task (Patro et al., in press). Furthermore, culture-dependent biases in line bisection (Chokron and De Agostini, 1995; Rinaldi et al., 2014) as well as a culture-dependent preferences for the direction of drawing (Kebbe and Vinter, 2013) have been reported for the horizontal direction. So it is plausible that culture-dependent preferences in performing motor actions (such as pointing) might have contributed to the counting bias. Future studies should investigate directional preferences for both counting and pointing.

In summary, I have discussed the evidence for grounded, embodied, and situated origins of horizontal and vertical SNAs. A *combination account* (de Hevia et al., 2012; Nuerk et al., 2015) is emerging: due to hemispheric lateralization (Rugani et al., 2014, 2015) and a preference for increasing magnitudes (Macchi Cassia et al., 2012) we start life with a slight preference to associate small magnitudes with the left side of space (a biological predisposition). In addition, interactions with the physical world (grounded cognition; Barsalou, 2008) lead us to expect magnitudes to increase from the bottom to the top resulting in an initial SNA with increasing magnitude from bottom left to top right. Interactions with cultural spatial biases in the environment such as exposure to cultural reading practices then modify this initial bias: depending on the culturally predominant spatial directionality the bottom left to top right bias either gets strengthened, weakened, or overwritten. Although further research into other cultural spatial biases is needed, current evidence favors reading direction as the strongest cultural spatial influence. SNAs molded by longstanding cultural directional biases can also be modified temporarily by recent spatial experiences.

To conclude, our findings clearly support the influence of primary and secondary reading direction on the horizontal and vertical direction of counting in the horizontal plane and its relationship to recent as well as longstanding reading exposure and experience.

## Conflict of Interest Statement

The author declares that the research was conducted in the absence of any commercial or financial relationships that could be construed as a potential conflict of interest.

## Footnotes

1 Many street and shop signs in HK are vertical. In a pilot study in 2012, 100 books for children and 100 books for adults were picked randomly from the shelves of the Hong Kong Central Library. Eighty percent of the books for children used a left-to-right reading direction, 17% a top-to-bottom reading direction, and 3% mixed reading directions. For adult books the corresponding percentages were: 46% left-to-right, 42% top-to-bottom, and 12% mixed.
