# Supplementary material for: Up or down? Reading direction influences vertical counting direction in the horizontal plane – a cross-cultural comparison
Source: Front Psychol. 2015 Mar 10;6:228. doi: 10.3389/fpsyg.2015.00228 (PMC4366652; doi:10.3389/fpsyg.2015.00228)
Supplement: Supplementary file 1 [file table_1.docx]

*Supplementary Table 1: Number of participants by counting direction for the horizontal and vertical displays for experiment 1*

|  | Horizontal display | | |  | | Vertical display | |
| --- | --- | --- | --- | --- | --- | --- | --- |
| Group | | Left to right | Right to left | |  | Bottom to top | Top to bottom |
| British | |  |  | |  |  |  |
| Children | | 41 | 30 | |  | 53 | 18 |
| Adults | | 84 | 6 | |  | 15 | 75 |
| HK-Chinese | |  |  | |  |  |  |
| Children | | 79 | 6 | |  | 16 | 69 |
| Adults | | 87 | 12 | |  | 13 | 86 |
