# Supplementary material for: Up or down? Reading direction influences vertical counting direction in the horizontal plane – a cross-cultural comparison
Source: Front Psychol. 2015 Mar 10;6:228. doi: 10.3389/fpsyg.2015.00228 (PMC4366652; doi:10.3389/fpsyg.2015.00228)
Supplement: Supplementary file 2 [file table_2.docx]

*Supplementary Table 2: Number of participants by starting position and direction of first movement for counting the square display for experiment 2*

|  | Starting position | | | | |  | | First movement | | | | | |
| --- | --- | --- | --- | --- | --- | --- | --- | --- | --- | --- | --- | --- | --- |
|  | Left | |  | Right | | |  | Horizontal | |  | Vertical | | |
| Group | top | bottom |  | top | bottom | |  | left-right | right-left | |  | bottom-top | top-bottom |
| Horizontal text |  |  |  |  |  | |  |  |  | |  |  |  |
| UK stay < 3y | 10 | 0 |  | 2 | 0 | |  | 10 | 0 | |  | 0 | 2 |
| UK stay > 3y | 33 | 0 |  | 0 | 0 | |  | 31 | 0 | |  | 0 | 2 |
| Vertical text |  |  |  |  |  | |  |  |  | |  |  |  |
| UK stay < 3y | 12 | 0 |  | 8 | 0 | |  | 12 | 0 | |  | 0 | 8 |
| UK stay > 3y | 15 | 0 |  | 7 | 0 | |  | 15 | 0 | |  | 0 | 7 |
