# Supplementary material for: Up or down? Reading direction influences vertical counting direction in the horizontal plane – a cross-cultural comparison
Source: Front Psychol. 2015 Mar 10;6:228. doi: 10.3389/fpsyg.2015.00228 (PMC4366652; doi:10.3389/fpsyg.2015.00228)
Supplement: Supplementary file 3 [file data_sheet_1.docx]

***Supplementary Material***

**Up or down?**

**Reading direction influences vertical counting direction –**

**a cross-cultural comparison**

**Silke M. Göbel^1^***

1 Department of Psychology, University of York, York, U.K.

*** Correspondence:** Silke M. Göbel, Department of Psychology, University of York, Heslington, York,YO10 5DD, U.K.

Silke.goebel@york.ac.uk

1. **Supplementary Data**

**Supplementary Material**

A: Horizontal text

**坦然面對**

①飛速行駛的列車上，一位老人剛買的新鞋不慎從視窗掉下去一隻。周圍的旅客無不為之惋惜，不料老人毅然把剩下的那只也扔了下去。眾人大惑不解，老人坦然一笑：“鞋無論多麼昂貴，剩下一隻對我來說就沒有什麼用處了。把它扔下去，就可能讓揀到的人得到一雙新鞋說不定他還能穿呢。”老人看似反常的舉動，體現了他清醒的價值判斷：與其抱殘守缺，不如果斷放棄。這種坦然面對失去的豁達心態，令人頓生敬意，也發人深思。

②一般來說，人們總是習慣於得到而害怕失去。儘管“有得必有失”的道理人人皆知，但人們依舊認為得到了可喜可賀，而失去則可惜可歎。每有所失，總要難受一陣，甚至為之痛苦。

③人生苦短。為了不虛度光陰，使生命盡可能卓越，我們的確應該追求得到，努力用智慧和汗水去創造業績。然而，我們也應該正確看待得失，學會忍受失去。為了成就一番事業，有時不得不失去一些感官的享受；為了更好地實現自己的主要人生目標，有時不得不“丟卒保車”；尤其是為了不玷污自己的人格，有時不得不失去一些利益，比如金錢——那種只要出賣良心或尊嚴就可以得到的金錢。

④坦然面對失去，需要及時調整心態，首先要面對現實，承認失去，不能沉湎於已經不存在的東西之中。得到和失去其實是相對的。為了得到，需要失去，因為失去一些，可能又意想不到地得到了另一些。民間安慰丟東西的人總是說：“舊的不去新的不來”。事實正是如此。（    ）為了失去而懊惱，（    ）全力爭取新的得到。應該明白的是，有時失去並不一定是損失，而是放棄，是奉獻，是大步躍進的前奏或序曲，這樣的失去，不也是好事麼？

⑤坦然面對失去，不是像有些人那樣自我姑息，也不是像某些人那樣“看破紅塵”，碌碌無為地苟活。坦然面對失去，就是胸襟更豁達一些，眼光更長遠一些，經常為自己整整枝、打打杈，排除那些不必要的留戀與顧盼，以便集中精力于人生的主要追求。這樣，大而言之，有益於社會；小而言之，有益於自己。

1.本文的中心論點是______________________________________________________。

2.第③、④段分別從哪些方面論述“坦然面對失去”？

__________________________________________________________________________________________________________________________________________________

3.“老人看似反常的舉動……”為什麼說老人的舉動反常？

__________________________________________________________________________________________________________________________________________________

4.“比如金錢——那種只要出賣良心或尊嚴就可以得到的金錢”。沒有破折號後面的一句話，文句也通順，為什麼還要加上這句話？

__________________________________________________________________________________________________________________________________________________

5．第④段的括弧中所填關聯詞，準確的一項是（  ）

A、如果 那麼    B、儘管 還是   C、與其 不如     D、雖然 但是

6．結合文中的觀點，簡要評析下面的兩種表現。

甲、乙兩支球隊力量相差懸殊，甲隊自知拼不過乙隊，就派非主力隊員與乙隊比賽，而將主力保存與另外的隊比賽。

 某人參加3000米長跑，與別人拉下一段距離，他便退出了。

__________________________________________________________________________________________________________________________________________________

__________________________________________________________________________________________________________________________________________________

**Supplementary Material**

B: Vertical text


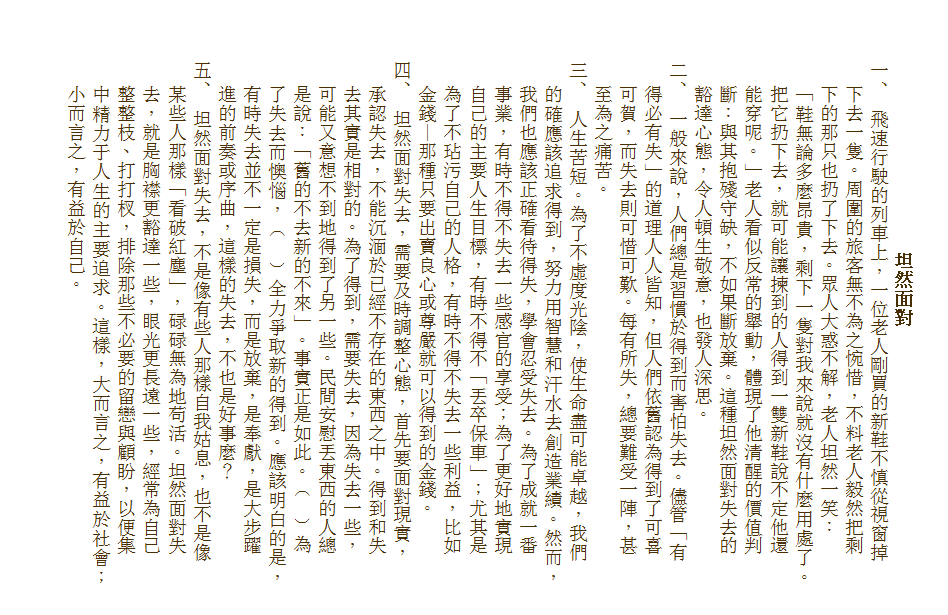


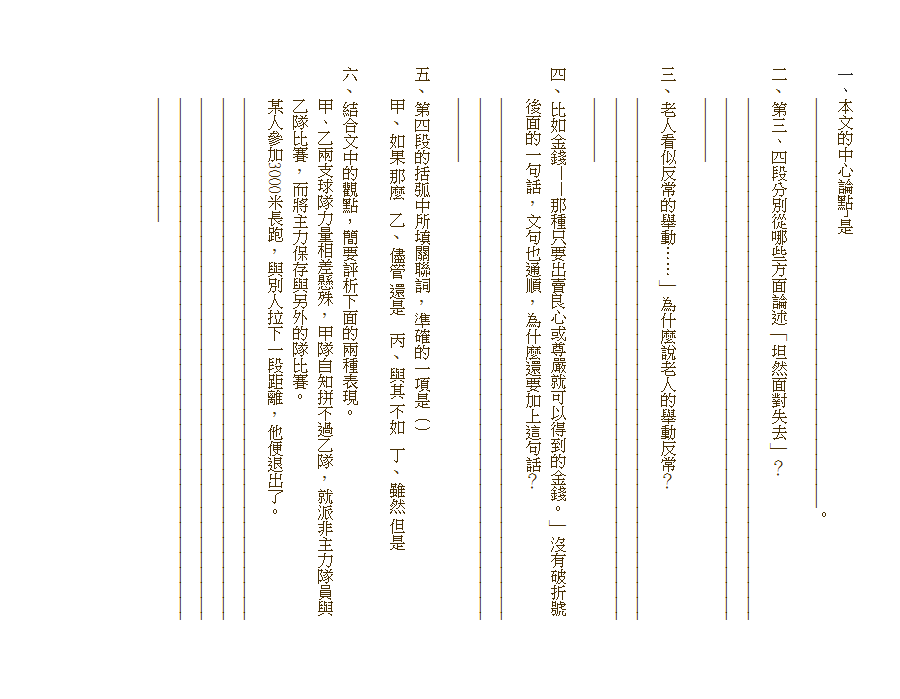


**Supplementary Material**

C: English translation

**Facing Loss with Equanimity**

(1) One day on a speeding train, one of an old man’s new shoes accidentally fell out of the window. The passengers around him all felt sorry for him. Unexpectedly, the old man threw the other shoe out of the window! While everyone was puzzled by his action, the old man smiled and explained, “No matter how expensive the shoes are, they are useless to me if I only have one of them. Now I have thrown the other shoe out, perhaps someone else may have the new pair of shoes and even find them wearable!” What the old man did might seem unusual, but it has reflected his clear-valued judgement; giving up rather than holding on meaninglessly. The way the old man faced loss with an open-mind was considered respectful and thought-provoking.

(2) In general, people prefer gaining and fear losing. Although everyone knows the concept of ‘if there’s a winner, there’s always a loser’, but it is still common practise to celebrate gain and mourn over loss.

(3) Life is short. In order not to waste time and make life as fulfilling as possible, we should always pursue accomplishment with wisdom and hard work. But, we should also have a correct view on gains and losses, such as learning to endure losses in our lives. In order to be successful in our careers, sometimes we have to give up certain pleasures. In order to better achieve the main goal in life, sometimes we have to protect the major things at the cost of the minor ones. In order to retain our dignity, sometimes we have to sacrifice certain benefits, such as money - the kind of money you can get from selling your conscience or self-respect.

(4) We need to adjust our attitudes toward losing. First of all, we have to face the reality and admit to losing, and not indulge in something that no longer exists. Gaining and losing are relative. We always have to lose something for the sake of getting something else. We may even get something unexpected through losing. There is an old Chinese saying: "If we cannot let go of the past, we cannot welcome the future.” This is indeed the truth. ( **Q5** ) being upset because of losing, (**Q5**) spend our effort to gain something new. It should be understood that sometimes letting go is not necessarily a loss, but to give up or dedicate, can be the beginning of another chapter. Is not that kind of losing also a good thing?

(5) Letting go is not like self-appeasement or living a meaningless life. Letting go, is taking a long-term vision with an open-mind. We should evaluate our lives constantly to exclude unnecessary nostalgia in order to concentrate on the main pursuit in life. Through this way, we can be beneficial to society and to ourselves.

1) The main argument of this article is ______________________________________________________.

2) In what aspect does paragraph 3 and 4 explain the idea of facing ‘lost’ with equanimity?

__________________________________________________________________________________________________________________________________________

3. "The elderly seems to act abnormally ..." Why do people think the behaviour of the elderly seems unusual?

__________________________________________________________________________________________________________________________________________________

4. "Such as money – those you can get as long as you betray your morality or dignity." The sentence is still fluent without the last sentence after the dash. Why did the author add this sentence? __________________________________________________________________________________________________________________________________________

5. Which of the below connective words suit best in the bracket of paragraph ④?

A. If...will B. Although C. Rather than…it is better D. …but…

6. By adopting the point of view from the above article, analyse the two attitudes below.

Team A and Team B has a significant difference in terms of strength. Team A knows that they will probably lose in the match. Therefore, they sent non-key players to play with Team B and let the anchors to participate in other matches.

Peter participates in a 3000 meter run. He withdraws himself from the competition since he is behind the others for a certain distance.

__________________________________________________________________________________________________________________________________________________________________________________________________________________________________________

1. **Supplementary Figures and Tables**

## Supplementary Tables

*Supplementary Table 1: Number of participants by counting direction for the horizontal and vertical displays for experiment 1*

|  | Horizontal display | | |  | | Vertical display | |
| --- | --- | --- | --- | --- | --- | --- | --- |
| Group | | Left to right | Right to left | |  | Bottom to top | Top to bottom |
| British | |  |  | |  |  |  |
| Children | | 41 | 30 | |  | 53 | 18 |
| Adults | | 84 | 6 | |  | 15 | 75 |
| HK-Chinese | |  |  | |  |  |  |
| Children | | 79 | 6 | |  | 16 | 69 |
| Adults | | 87 | 12 | |  | 13 | 86 |

*Supplementary Table 2: Number of participants by starting position and direction of first movement for counting the square display for experiment 2*

|  | Starting position | | | | |  | | First movement | | | | | |
| --- | --- | --- | --- | --- | --- | --- | --- | --- | --- | --- | --- | --- | --- |
|  | Left | |  | Right | | |  | Horizontal | |  | Vertical | | |
| Group | top | bottom |  | top | bottom | |  | left-right | right-left | |  | bottom-top | top-bottom |
| Horizontal text |  |  |  |  |  | |  |  |  | |  |  |  |
| UK stay < 3y | 10 | 0 |  | 2 | 0 | |  | 10 | 0 | |  | 0 | 2 |
| UK stay > 3y | 33 | 0 |  | 0 | 0 | |  | 31 | 0 | |  | 0 | 2 |
| Vertical text |  |  |  |  |  | |  |  |  | |  |  |  |
| UK stay < 3y | 12 | 0 |  | 8 | 0 | |  | 12 | 0 | |  | 0 | 8 |
| UK stay > 3y | 15 | 0 |  | 7 | 0 | |  | 15 | 0 | |  | 0 | 7 |
